# Effects of Flavonoid-Rich Orange Juice Intervention on Major Depressive Disorder in Young Adults: A Randomized Controlled Trial

**DOI:** 10.3390/nu15010145

**Published:** 2022-12-28

**Authors:** Jihee Choi, Jong-Hoon Kim, Miey Park, Hae-Jeung Lee

**Affiliations:** 1 Department of Food and Nutrition, College of BioNano Technology, Gachon University, Seongnam-si 13120, Republic of Korea; 2 Institute for Aging and Clinical Nutrition Research, Gachon University, Seongnam-si 13120, Republic of Korea; 3 Department of Psychiatry, Gachon University College of Medicine, Gil Medical Center, Neuroscience Research Institute, Gachon University, Incheon 21565, Republic of Korea; 4 Department of Psychiatry, Gachon University College of Medicine, Gil Medical Center, Incheon 21565, Republic of Korea

**Keywords:** flavonoid therapy, major depressive disorder, randomized controlled trial, *Butyricicoccus pullicaecorum*

## Abstract

Many individuals are suffering from depression, and various improvements are being proposed. This study was conducted on young people diagnosed with depression and aimed to assess the effects of flavonoid-rich orange juice on the major depressive disorder (MDD) using a randomized controlled trial. In all, 40 young men and women with MDD aged 18–29 years were randomly assigned to a flavonoid-rich orange juice group (FR group) and a flavonoid-low orange cordial group (FL group). The subjects drank the corresponding juice three times a day (190 mL per bottle) for 8 weeks. The blood BDNF, zonulin, and claudin-5 levels significantly increased (*p* < 0.0001, *p* < 0.01, and *p* < 0.05, respectively) in the FR group, and the fatty acid binding protein 2 (FABP2) level was significantly decreased (*p* < 0.0001) in the FR group after the juice intervention. The FABP2, LPS, and valeric acid levels were negatively correlated with the abundance of *Butyricicoccus pullicaecorum*, which was higher in the FR group. Orange juice intake improved depressive symptoms in young adults with MDD in the FR group. This *B. pullicaecorum* can be a potential biomarker for clinical improvement in young adults with MDD patients.

## 1. Introduction

Major depressive disorder (MDD) is a prevalent mental health disorder worldwide that severely restricts the patient’s psychosocial functions and reduces their quality of life [1,2]. Typically, MDD persists for at least two weeks, and refers to mood disorders with at least five clinical symptoms, such as decreased motivation or interest, reduced self-esteem, severe weight gain or loss, increased fatigue, decreased concentration, insomnia, and suicidal thoughts [3,4]. Therefore, if MDD becomes chronic and untreated, it can interfere with daily or social life.

Combinations of drugs, such as antidepressants and psychotherapy, are the most effective approaches to treating MDD [5]. However, antidepressants are found to be associated with adverse effects [6]. Given the psychotherapy effects of several herbal remedies, herbal remedies may be a promising and practical approach to treating depression [7]. Recent systematic reviews and meta-analyses revealed that flavonoids have a high potential for reducing inflammation and oxidative stress, which makes them helpful in alleviating the symptoms of depression and anxiety [8,9]. Some of the possible effects of flavonoids on neurotransmitters are increased brain-derived neurotrophic factor (BDNF) signaling, reduced oxidative stress, increased neurogenesis, and synaptic plasticity, increased monoamine neurotransmitters, and decrease neuroinflammation [10].

Serotonin (5-hydroxytryptamine, 5-HT) is a monoamine neuromodulatory transmitter that has an essential physiological function in conjunction with catecholamines such as dopamine and norepinephrine. Serotonin has unique neuroplastic functions, and synaptic plasticity is a critical mechanism in learning and memory. Dysfunction of synaptic plasticity could induce the development of MDD, induce neuronal atrophy, and ultimately cause cell death [11,12,13,14]. According to recent studies on synaptic plasticity mechanisms, GABAergic interneurons inhibit other neurons in the cortex to coordinate cortical activity and modulate synaptic plasticity [15]. Defects in the GABAergic neurotransmitter system could cause MDD [15]. However, it is still only a hypothesis, and it seems to be a topic we need to check in the future [16].

BDNF is a common neurotrophin in the central nervous system (CNS). It is a nerve function regulator involved in the survival and differentiation of nerve cells, synaptic transmission, and synaptic plasticity. Because an antidepressant selective serotonin reuptake inhibitor (SSRI) increases BDNF gene expression, BDNF is thought to be a biomarker for MDD [17]. Insight into the mechanism of MDD has facilitated the development of novel pharmaceuticals with greater efficacy and fewer side effects. However, only 40–70% of MDD patients respond to antidepressant treatments [18,19]. Therefore, it is important to come up with novel treatments and add-on therapies for people with MDD [20].

The psychobiomes that exert a clinical effect through endocrine agents associated with the control of neuroinflammation and the generation of neurotransmitter precursors are identified by the correlations between modified gut microorganisms and MDD [21,22]. Psychological stresses affect the composition of the gut microbiome via this pathway. However, the state of the gastrointestinal tract also affects brain function [23,24,25]. Intestinal bacteria can affect diseases of the central nervous system, and the interaction between intestinal bacteria and the human nervous system can be achieved through the control of the host’s neurotransmitters or related pathways. Bacteria have been found to have the ability to produce significant neurotransmitters [26]. For example, *Lactobacillus Plantarum* produces serotonin, histamine [27], and acetylcholine [28]. GABA is released by Bifidobacterium [29] and Bacillus can release dopamine and noradrenaline [30]. In addition, many studies have shown that SCFA contributes to the maintenance of physical barriers such as blood–brain barriers (BBBs) or intestinal barriers by influencing close bonding between cells [31,32]. The permeability of BBBs is affected by inflammatory cytokines caused by lipopolysaccharides (LPS) produced by bacteria [33].

It is possible to alter the gut microbiota–brain axis and reduce the occurrence of MDD via the diet by altering the gut microbiota profile and regulating intestinal permeability [34]. Consuming enough polyphenols, such as flavonoids, and eating fruits and vegetables can reduce the risk for MDD [35,36,37]. This is not due to the direct effect of dietary polyphenols but to the metabolites of polyphenols by intestinal microorganisms [22,38]. Improvements are caused via the gut microbiota–brain axis as a result of dietary polyphenols [22,38]. Cognitive function is improved by polyphenols in citrus fruits, although some clinical studies have ignored the role of sufficient flavonoid intake in improving MDD symptoms [39,40,41]. Therefore, using a single-blind randomized controlled trial, we looked at the microbiota profile and MDD symptoms in 40 young adults with MDD who had never taken an antidepressant before and who drank orange juice, which is one of the most common sources of flavonoids.

## 2. Materials and Methods

### 2.1. Study Design

We evaluated whether consuming orange juice containing flavonoids improved MDD symptoms in a single-blind, randomized controlled trial of 8-week duration. The trial was registered with the Gachon University Institutional Review Board (IRB, 1044396-201904-HR-066-01). The study was conducted following the ethical principles for medical research involving human subjects stipulated in the Declaration of Helsinki. Written informed consent was obtained from all participants after they had received explanations of its goals, test schedule, and potential risks. The study protocol was registered with the Clinical Research Information Service (https://cris.nih.go.kr/cris/index/index.do (accessed 13 May 2021), KCT0006137), and protocol modifications were publicly reflected through CRIS. The participants visited the Neuroscience Research Institute of Gachon University and underwent the mini-international neuropsychiatric interview plus (MINI-Plus), including clinical assessments for MDD, conducted by a professional clinical psychologist one week before the commencement of the study, to confirm whether they met the inclusion criteria. Subjects with MDD were randomly assigned to a flavonoid-rich orange juice (FR) or flavonoid-low orange cordial (FL) group using random numbers. Treatment drinks were provided in the order of registration based on a randomized list. Subjects were instructed to consume FR or FL three times a day (30–60 min before breakfast, lunch, and dinner; 190 mL per bottle) for 8 weeks [39]. Baseline data, including the subjects’ sex, age, and medical history, were obtained during MDD assessments. Before the intervention, the participants were also evaluated for lifestyle factors, including income, drinking, smoking, and stress levels. The subjects underwent a 24 h dietary recall, gut microbiome analysis (GMA), blood tests, and anthropometric measurements on the first treatment day (baseline), 1 week after the MDD assessment visit, and at the end of treatment (8 weeks later). The subjects were blinded to the treatment allocation until the study was finished.

### 2.2. Participants

We estimated the minimum sample size through power calculations, and the estimated minimum sample size for each group was 17. We aimed to recruit 40 subjects (20 subjects per group), with an estimated dropout rate of 20%. Accordingly, we recruited male and female adults aged 18–29 years who lived in the Incheon and Gyeonggi-do regions of South Korea from June to December 2019. The participants (*n* = 40) provided written consent to participate in the study.

Inclusion criteria: The participants were aged 18–29 years and diagnosed with MDD based on the Beck Depression Inventory-II (BDI-II) [42], Hamilton Depression Rating Scale (HAMD-17) [43], and Center for Epidemiological Studies Depression Scale (CES-D) [44], which are the most widely used MDD screening tools. The diagnosis of MDD was determined using the MINI-Plus, a structured diagnostic interview tool. A Psychiatrist conducted the tests.

Exclusion criteria: We excluded individuals with a family history of psychiatric disorders, a previous diagnosis of psychiatric disorders, a history of using psychotropic drugs and antibiotics, or a bowel disease. In addition, subjects with other psychiatric diagnoses were excluded, and subjects requiring urgent inpatient treatment or immediate pharmacotherapy were also excluded from the study.

### 2.3. MDD Assessments

Beck Depression Inventory-II (BDI-II): The BDI-II was developed by Beck et al. [45] to measure the severity of depressive symptoms. It includes 21 items that encompass cognitive, emotional, motivational, and physical symptoms to measure depressive symptoms. It uses responses (on a 4-point Likert scale) to questions to assess the severity of depressive symptoms. Depressive symptom scores range from 0 to 63, with higher scores indicating more severe depressive symptoms. Severity is generally classified as non-depression (0–9), mild depression (10–15), moderate depression (16–23), or severe depression (24–63). We enrolled subjects with BDI-II scores ≥ 23 [42].

Center for Epidemiological Studies Depression Scale (CES-D): The CES-D was developed by Radloff [44] to evaluate depressive symptoms. It is an MDD screening tool that measures symptom severity based on their duration. In addition, it enables screening of high-risk groups and assessment of the correlations between depressive symptoms and other variables, and its effectiveness (e.g., consistency) has been validated. We placed subjects with scores ≥ 21 into the MDD group [46].

Hamilton Depression Rating Scale (HAMD-17): The HAMD-17 includes a depressed mood, feelings of guilt, suicidal thoughts, work and activities, retardation, agitation, psychic anxiety, somatic anxiety, hypochondriasis, insomnia (early), insomnia (middle), insomnia (late), gastrointestinal somatic symptoms, general somatic symptoms, weight loss, genital symptoms, and insight. It is not a self-report, such as the BDI, MDQ, and CES-D, but an assessment tool that enables clinicians or clinical psychologists to evaluate MDD objectively. The depressive symptom score ranges from 0 to 52, with higher scores indicating more severe depressive symptoms. Severity is divided into normal (0–7), mild depression (8–13), moderate depression (14–18), severe depression (19–22), and very severe depression (≥23). We categorized subjects with scores ≥ 14 into the MDD group [47].

### 2.4. Treatment Drinks

We used commercially available orange juice as a treatment drink based on the recommendation of experts that patients with depression could experience side effects compared to healthy people after consuming highly concentrated orange extracts. We used 100% pure Florida orange juice (Natalie’s Orchid Island Juice Co., FL, USA and Tomato Agricultural Association Corporation Inc., Paju-si, Republic of Korea; 190 mL) in the FR group and orange-flavored cordial (Del Monte Foods Inc., CA, USA and Lotte Chilsung Beverage Co., Ltd., Seoul, Republic of Korea; 190 mL) in the FL group. The complete flavonoid contents of FR and FL were analyzed by liquid chromatography-mass spectrometry (LC-MS). The total flavonoid content in FR was 157.9 ± 1.4 mg/100 g, and that in FL was 28.4 ± 0.7 mg/100 g (Supplementary Table S1). The two drinks had almost identical nutritional components, such as color, volume, calories, glucose, and fructose, but their flavonoid contents differed (Supplementary Table S2).

### 2.5. Blood Parameters

The subjects were instructed not to consume food or drinks other than water from 12 h before the blood test and were advised not to take any drug or alcohol for at least 24 h before the test. Blood samples (5 mL) were collected at the beginning of treatment (baseline) and at the end of treatment (after 8 weeks). BDNF, serotonin, folate, homocysteine, high-sensitivity C-reactive protein (hs-CRP), and vitamin B12 levels were analyzed in serum. Blood parameters were analyzed using SQLab (SQLab Co., Yongin, Republic of Korea).

### 2.6. Dietary Assessment

A trained interviewer conducted a 24 h recall questionnaire at the beginning (baseline) and end (after 8 weeks) of the treatment to evaluate food intake. Subjects were advised to maintain their usual food intake during the test period and instructed to refrain from consuming citrus fruits, dietary supplements, and drugs that could affect their flavonoid content and intestinal microbial flora. Food intake was evaluated using a computer-aided nutritional analysis program for experts (CAN-Pro 5.0, Korean Nutrition Society, Seoul, Republic of Korea). The subjects were also advised to maintain their usual physical activity during the test period.

### 2.7. Fecal Sample Collection and Quantifications of Fecal Short-Chain Fatty Acids

The subjects provided fecal specimens before (Before flavonoid-rich orange juice group (BFR), *n*= 20; Before flavonoid-low orange cordial group (BFL), *n* = 20) and after (After flavonoid-rich orange juice group (AFR), *n* = 20; After flavonoid-low orange cordial group (AFL), *n* = 20) the 8-week treatment. After collecting a fecal sample using a stool kit, fecal samples were stored at −80 °C until analysis. The fecal samples were added to 4 mL ice-cold MeOH with 1% formic acid per gram of feces, homogenized for 30 s, vortexed for 30 min, and stored at 4 °C for 2 h. After centrifugation (14,000 rpm) at 4 °C for 30 min, the supernatant was passed through a PVDF filter (0.22 μm). Next, 20 µL 3-nitrophenylhydrazine (3-NPH) and 20 µL N-(3-dimethylaminopropyl)-N-ethyl carbodiimide (EDAC) were added to 40 µL samples, vortexed for 1 min, then reacted at 40 °C, 30 min. Six short-chain fatty acids (SCFAs) were analyzed by LC-MS/MS (AB SCIEX, Framingham, MA, USA) using a sample volume of 1 μL and a column flow rate of 0.35 m/min at 40 °C.

### 2.8. Biochemical Assays

Serum was split into 500 μL aliquots to measure BDNF (R&D Systems, Minneapolis, MN, USA), zonulin (Human Zonulin ELISA Kit, Elabscience, Bethesda, MD, USA), lipopolysaccharide (LPS, LSBio, Seattle, WA, USA), Fatty Acid Binding Protein 2 (FABP2, Human FABP2 Quantikine ELISA Kit, R&D Systems, Minneapolis, MN, USA), and claudin-5 (Human Claudin-5 ELISA Kit, Novus Biologicals, Littleton, CO, USA).

### 2.9. PCR and Sequencing

The PCR first amplification of the V3 and V4 regions of 16S rRNA was performed using 341F (5′ TCGTCGGCAGCGTC-AGATGTGTATAAGAGACAG-CCTACGGGNGGCWGCAG-3′) and 805R (5′ GTCTCGTGG GCTCGG-, AGATGTGTATAAGAGACAG-GACTACHVGGGTATCTAATCC-3′) primers, as described previously [48]. The second PCR amplification for Illumina NexTera barcodes attachment was performed. Bioanalyzer 2100 (Agilent, Palo Alto, CA, USA) was employed to evaluate quality and product size using a DNA 7500 chip. Sequencing was performed in accordance with the instructions of the Illumina MiSeq Sequencing System (Illumina Inc., SD, USA).

### 2.10. Gut Microbiome Sequencing Data Analysis

Ribosomal 16S amplicon sequences were processed and analyzed using the newly updated pipeline for quantitative insights into microbial ecology and microbiome analysis (QIIME2 version 2020.11) [49]. The 80 pieces of paired-end reads were imported and used the quality control divisive amplicon denoising algorithm 2 (DADA2) for denoising, trimming, removing low-quality reads, and filtering chimeras [50]. According to the quality score visualization, different locations were used and retain the optimal read length and count in the DADA2 analysis. Furthermore, an amplicon sequence variants (ASVs) was completed and adopted for downstream studies from the filtered and non-chimeric sequences. A phylogenetic analysis was performed to generate a masked, rooted phylogenetic tree from the multiple sequence alignment (MSA) of all selected ASVs. MSA was performed using mafft [51], and the phylogenetic tree was constructed using FastTree [52]. Taxonomic classification of all ASVs/features was performed using a naïve Bayes taxonomy classifier trained on the Greengenes 13_8.99% OTUs [53]. Alpha diversity indices (Chao-1, Simpson, and Shannon diversity) were calculated using BIOiPLUG, ChunLab’s bioinformatics cloud platform.

### 2.11. Differential Abundance of Taxa

The differential abundance of taxa was calculated in the three comparisons by linear discriminant analysis effect size (LEfSe) analysis [54]. Level 7, i.e., the species-level collapsed table converted into the biome file format, was used for differential abundance analysis. LEfSe analysis was performed after an appropriate format change, using a galaxy cloud-based service. Cladograms and bar plots were generated to visualize the LEfSe results of differentially abundant taxa.

### 2.12. Statistical Analysis

Statistical analysis was performed using SAS software (version 9.4; SAS Institute, Inc.; Cary, NC, USA). Data are presented as means ± standard errors (SE) or frequencies. Differences in the dietary assessment before and after the 8-week treatment were determined by paired *t*-test and between groups with changes before and after treatment by two-sample *t*-test or Wilcoxon signed-rank test. Spearman’s rank correlation analysis was used to investigate the correlations between the gut microbiota and the clinical parameters of depressive symptoms and calculate the correlation coefficient (r) between the intestinal microflora and biomarkers. Statistical significance was determined at *p* < 0.05 and *p* < 0.01. R software version 4.1.3 was employed for Pearson correlation analysis of differences in plasma concentrations before and after intervention in the FL group.

## 3. Results

### 3.1. Subjects and Baseline Characteristics

**Figure 1:**
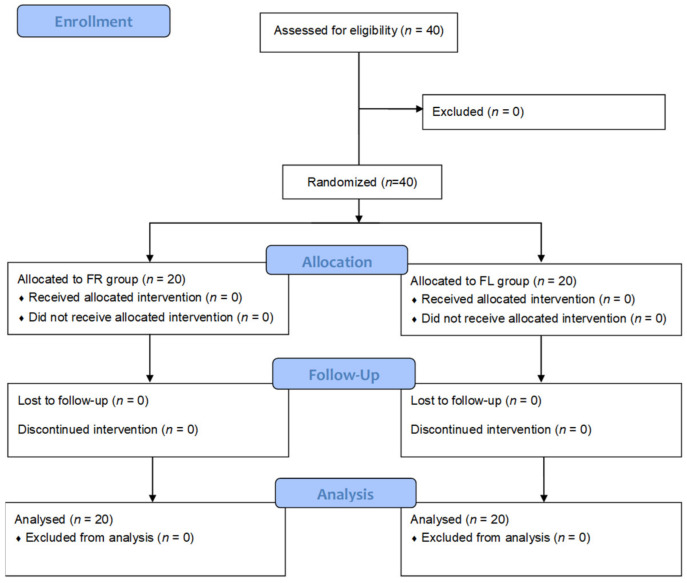
CONSORT flow diagram. FR group: flavonoid-rich orange juice group; FL group: flavonoid-low orange cordial group.

Forty subjects were randomly divided into the FR and FL groups (*n*= 20 each). The 40 subjects completed the test (Figure 1). The mean age of the subjects was 23.9 ± 2.71 years, and there were 12 and 28 male and female subjects, respectively. The baseline demographic characteristics of the subjects, including sex, age, residence type, household income, regular physical activity, alcohol drinking, heavy drinking frequency, smoking, and self-reported stress scales, were not significantly different between the FR and FL groups (Table 1).

### 3.2. Anthropometric Data, Blood Parameters, and MDD Scores

**Figure 2:**
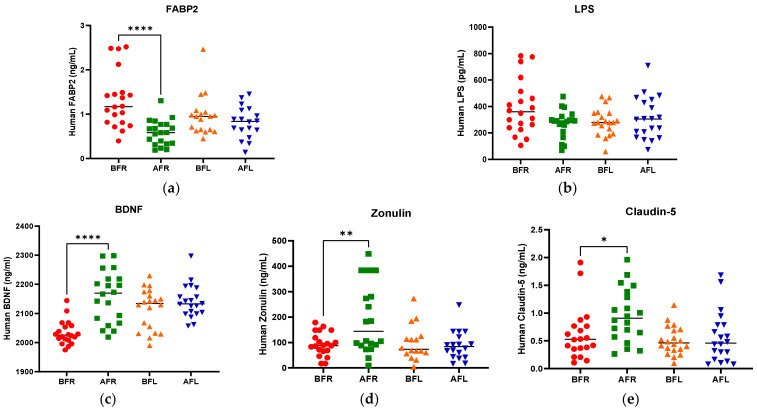
Difference between flavonoid-rich orange juice group or flavonoid-low orange cordial group in plasma levels before and after intervention. (**a**) fatty acid-binding protein-2 (FABP2) (**b**) LPS endotoxin (**c**) BDNF (**d**) Zonulin (**e**) Claudin-5. After 8 weeks of orange juice intervention, the blood markers of human FABP2 in the flavonoid-rich group decreased significantly compared to those of the flavonoid-low groups. In contrast, the BDNF and Zonulin in the flavonoid-rich group were increased significantly compared to those in flavonoid-low groups. BFR: Before flavonoid-rich orange juice group, AFR: after flavonoid-rich orange juice group, BFL: before flavonoid-low orange cordial group, AFL: after flavonoid-low orange cordial group. * *p* < 0.05; ** *p* < 0.01; **** *p* < 0.0001.

**Figure 3:**
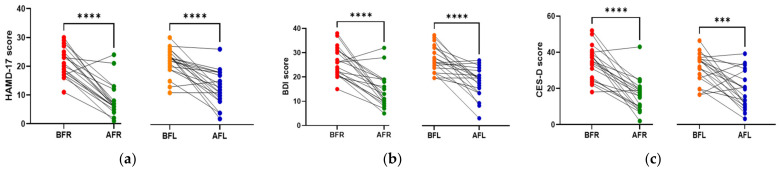
Differences between flavonoid-rich orange juice group or flavonoid-low orange cordial group before and after the intervention. (**a**,**b**) Post-intervention, the HAMD-17 and BDI-II scores in the AFR and AFL groups decreased significantly (*p* < 0.0001) compared to those of the BFR and BFL groups. (**c**) CES-D scores in the AFR (*p* < 0.0001) and AFL (*p* < 0.001) decreased significantly compared to those of the BFR and BFL groups. BFR: Before flavonoid-rich orange juice group, AFR: after flavonoid-rich orange juice group, BFL: before flavonoid-low orange cordial group, AFL: after flavonoid-low orange cordial group. BDI-II, Beck Depression Inventory-II; CES-D, Center for Epidemiological Studies Depression Scale; HAMD, Hamilton Depression Rating Scale. *** *p* < 0.001; **** *p* < 0.0001.

The anthropometric data, blood parameters, and MDD scores of the subjects are presented in Table 2. The body weight and body mass index (BMI) of both groups significantly increased (*p* < 0.05) during the test period; however, there were no significant between-group differences. Body fat, muscle mass, and blood pressure did not significantly change in either group during the test period. The BDNF level (*p* < 0.05) increased only in the FR group during the test period, and it was significantly (*p* < 0.05) different between the two groups. Although the two groups did not exhibit a significant difference in serotonin levels, the FL group’s level was found to be higher after 8 weeks (Figure 2). We assumed that depression is associated with human intestinal disorders and microbiota, which secrete toxins into the plasma and impair intestinal barrier integrity. We analyzed serum biomarkers in the 80 subjects before (BFR, *n* = 20; BFL, *n* = 20) and after (AFR, *n* = 20; AFL, *n* = 20) flavonoid treatment. The serum FABP2 level was significantly decreased (*p* < 0.0001) in the AFR relative to BFR groups. A reducing trend was observed in the AFL group compared to BFL groups without significance (Figure 2). The LPS level nonsignificantly decreased in the AFR versus the BFR groups. The BDNF (*p* < 0.0001) and zonulin (*p* < 0.01) levels were significantly increased in the AFR versus BFR groups compared to the AFL versus BFL groups. Furthermore, the level of claudin-5, a tight junction protein with neurological functions, was significantly elevated (*p* < 0.05) in the AFR versus BFR groups compared to the BFL group. The HAMD-17 (*p* < 0.0001), BDI-II (*p* < 0.0001), and CES-D (*p* < 0.001) scores of the two groups significantly decreased, indicating an improvement in MDD scores (Figure 3). Although there were no significant differences in MDD scores between the two groups, the decrease in symptom scores of the FR group over 8 weeks from baseline was numerically greater than that of the FL group.

### 3.3. Dietary Assessment

As shown in Supplementary Table S2, following the intervention, the values for energy, carbohydrate, protein, calcium, phosphate, iron, potassium, vitamin A, β-carotene, thiamin, riboflavin, niacin, pyridoxine, folate, and vitamin C intake in the FR group significantly increased compared to baseline (*p* < 0.05). This suggests an improvement in overall nutritional status. In the FL group, carbohydrate, potassium, thiamine, pyridoxine, folate, and vitamin C intake significantly increased (*p* < 0.05) compared to baseline. The intake of energy, carbohydrate, calcium, potassium, β-carotene, thiamine, pyridoxine, folate, and vitamin C differed significantly (*p* < 0.05) between the FR and FL groups (Supplementary Table S3).

### 3.4. Fecal Short Chain Fatty Acids (SCFAs)

**Figure 4:**
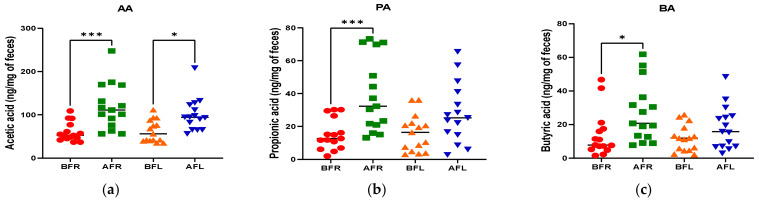

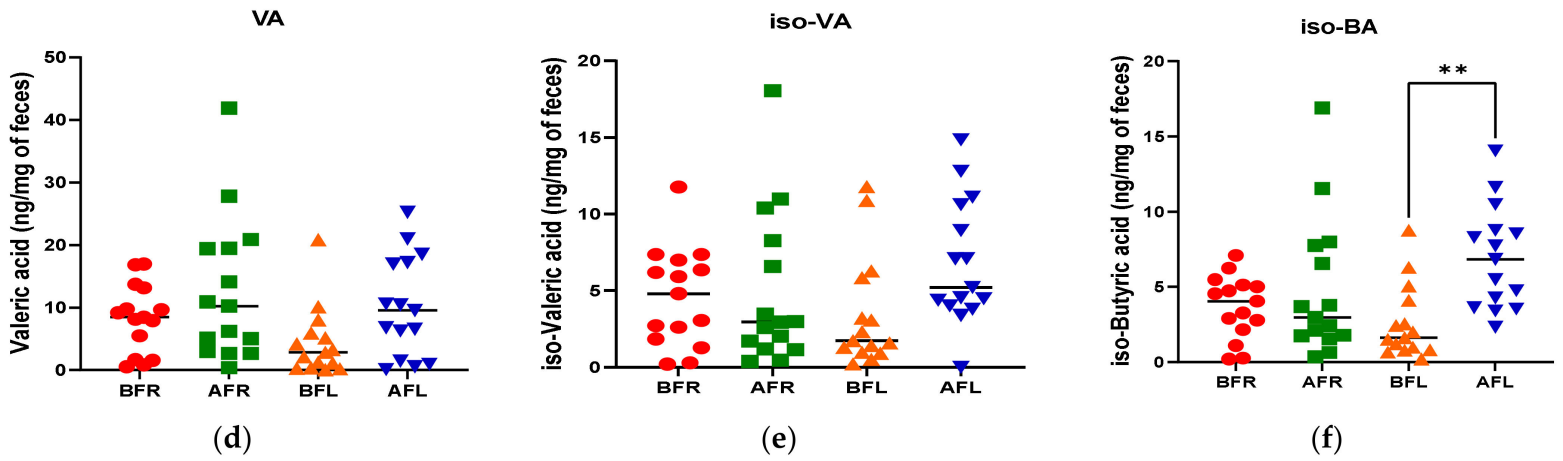
Differences between flavonoid-rich orange juice group or flavonoid-low orange cordial group in short-chain fatty acids (SCFAs) from before and after intervention with flavonoid-rich (BFR & AFR) or flavonoid-low (BFL & AFL) orange juice. AA: acetic acid (**a**), PA: propionic acid (**b**), BA: butyric acid (**c**), VA: valeric acid (**d**), iso-VA: iso-valeric acid (**e**), iso-BA: iso-butyric acid (**f**). BFR: Before flavonoid-rich orange juice group, AFR: after flavonoid-rich orange juice group, BFL: before flavonoid-low orange cordial group, AFL: after flavonoid-low orange cordial group. * *p* < 0.05; ** *p* < 0.01; *** *p* < 0.001.

The quantitative differences in acetic (*p* < 0.001), butyric (*p* < 0.05), and propionic (*p* < 0.001) acid levels between the BFR and AFR groups were significantly greater than those between the BFL and AFL groups. However, the quantitative difference in the isobutyric acid (*p* < 0.01) level between the BFL and AFL groups was substantially higher than that between the BFR and AFR groups (Figure 4).

### 3.5. Gut Microbiome Diversity

The Chao1, Simpson, and Shannon diversity indices were used to evaluate gut microbial diversity (Supplementary Figure S1). Regarding Faith’s phylogenetic diversity, alpha diversity slightly decreased in the AFR group compared to the BFR and AFL groups. Similarly, the alpha diversity of the AFL group was slightly lower than that of the BFL group. However, these changes were not significant. Beta diversity did not differ significantly (*p* = 0.929 for the AFL and BFL groups, *p* = 0.931 for the AFR and BFR groups, and *p* = 0.657 for the AFL and AFR groups) between the groups by permutation multivariate analysis of variance. Furthermore, no clear pattern of beta diversity was detected in the three comparisons based on Bray–Curtis distance-based principal coordinate analysis (PCoA) plots (Supplementary Figure S2).

### 3.6. Abundance of Bacterial Taxa

**Figure 5:**
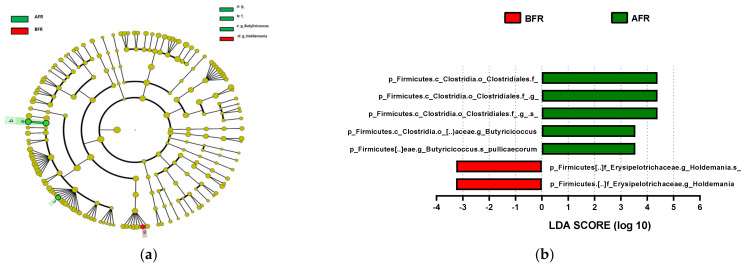

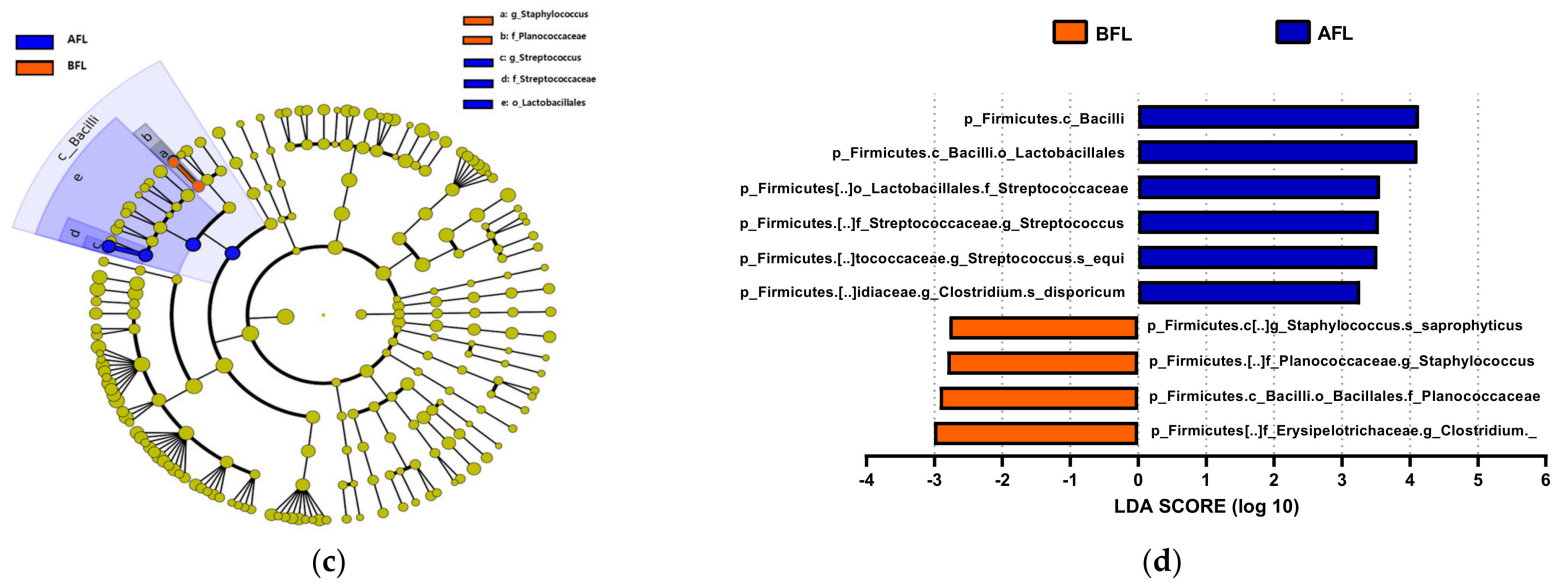
Differences between the taxonomic composition of before and after flavonoid-rich orange juice group and flavonoid-low orange cordial group intervention in depression. Lefse (**a**,**c**) and LDA analysis score (**b**,**d**) show the difference in abundance of bacterial taxa between before and after intervention with flavonoid-rich orange juice groups. BFR: Before flavonoid-rich orange juice group, AFR: after flavonoid-rich orange juice group, BFL: before flavonoid-low orange cordial group, AFL: after flavonoid-low orange cordial group.

**Figure 6:**
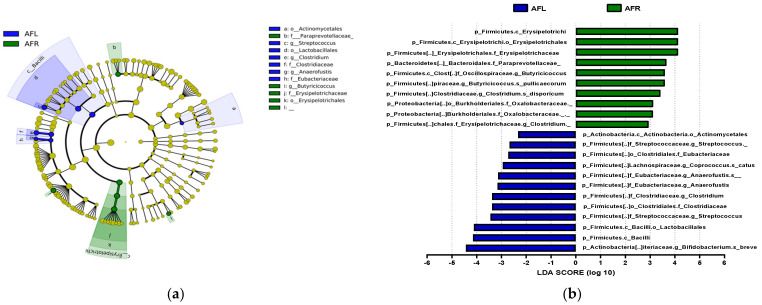
Differences between the taxonomic composition of after flavonoid-low orange juice group and flavonoid-rich orange cordial group intervention in depression. Lefse (**a**) and LDA analysis score (**b**) present the differences in the abundance of bacterial taxa after intervention between the flavonoid-low orange juice and flavonoid-rich orange cordial groups, respectively. AFL: after flavonoid-low orange cordial group, AFR: after flavonoid-rich orange juice group.

The predominant phylum in the four groups was Firmicutes, followed by Bacteroidetes and Actinobacteria (Figure 4 and Supplementary Figure S1). LEfSe analysis revealed that 7, 10, and 22 phyla were differentially abundant between the AFR/BFR, AFL/BFL, and AFR/AFL groups, respectively (Figure 5). *B. pullicaecorum* abundance in the AFR group was enhanced in the AFR/BFR and AFR/AFL comparisons (Figure 5 and Figure 6).

### 3.7. Correlations of Differences before and after Interventions in the FR Group

**Figure 7:**
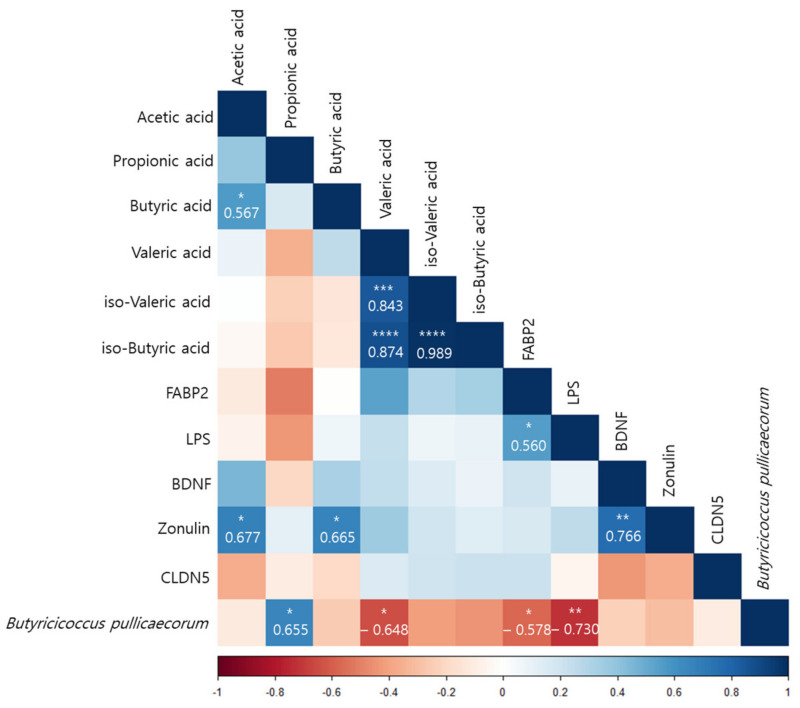
Pearson correlation analysis presenting the correlation between *Butyricicoccus pullicaecorum* and the plasma concentrations and the amount of fecal SCFAs before and after intervention in the flavonoid-rich orange juice group. After 8 weeks of orange juice intervention, *Butyricicoccus pullicaecorum* significantly negatively correlated with the blood markers of human FABP2, LPS, and valeric acid and was significantly positively correlated with the amount of propionic acid. * *p* < 0.05; ** *p* < 0.01; *** *p* < 0.001; **** *p* < 0.0001.

According to a Pearson correlation analysis of the orange juice group, *B. pullicaecorum* was significantly correlated with the propionic acid (r = 0.655, *p* < 0.05), valeric acid (r = −0.648, *p* < 0.05), FABP2 (r = −0.578, *p* < 0.05), and LPS (r = −0.730, *p* < 0.01) levels (Figure 7).

## 4. Discussion

Depression is related to eating habits [55,56]. The subjects in this study had poor nutritional status at baseline; however, their nutritional status improved as their MDD symptoms improved during the test period (Supplementary Table S3). Li et al. [57] and Lee et al. [47] reported that a high intake of red meat, processed meats, refined grains, high-fat dairy products, and sugars, together with a low intake of fruits and vegetables, increases the risk for depression. Moreover, Li et al. [57], Hintikka et al. [58], Logan [59], and Khalid et al. [60] demonstrated that the consumption of fruits, vegetables, unrefined whole grains, olive oil, fish, low-fat dairy products, tea, and foods rich in omega-3 fatty acids, zinc, folic acid, and flavonoids was significantly associated with a reduced incidence of depressive disorders. Notably, high levels of dietary flavonoids lower the risk for depressive disorders [35,36,37,61]. Flavonoids are polyphenols that are abundant in fruits, vegetables, and teas, their consumption increases the amount of blood delivered to the CNS, improving brain function [41,62,63,64,65,66,67]. Flavonoids consumed orally modulate the intestinal microbial flora, which affects not only flavonoid activity but also the level of tryptophan, increasing the synthesis of serotonin (5-hydroxytryptamine, a neurotransmitter), which ameliorates depression [68,69,70,71,72,73]. In this study, MDD status improved after both interventions. Although the serotonin level increased in the FR group, there were no differences between the FR and FL groups. However, the serum FABP2 level significantly decreased (*p* < 0.0001) in the FR group compared to the FL group. The FR group also exhibited more significant changes in serum BDNF (*p* < 0.0001), zonulin (*p* < 0.01), and claudin-5 (*p* < 0.05). FABP2 is a major biomarker associated with gut cell death, and a decrease in serum concentration means less damage to intestinal cells by the FR group than the FL group. Additionally, the zonulin and claudin-5 are associated with regulating tight junctions in the gut and BBB transfer and appear to recover more than in the FR group. Therefore, the flavonoid-rich orange supplement was more effective in terms of increasing the level of BDNF, which affects brain signaling and synaptic plasticity [74]. This indicates that such supplements influence the interaction between the intestinal microbiome and BDNF. The flavonoid-rich orange supplement induced a significant increase in the acetic (*p* < 0.001), propionic (*p* < 0.001), and butyric (*p* < 0.05) acid levels in patients with MDD. The increase in the FR group of acetic, propionic, and butyric acid, major SCFAs of intestinal microbial metabolism, is also associated with increased intestinal serotonin production and improved depression. SCFA, produced in the colon by the bacterial fermentation of resistant starch, etc., is implicated in controlling the neuroimmune endocrine system [75]. Acetate, propionate, and butyrate are the most abundant SCFAs in the gastrointestinal tract [76]. Although humans lack the enzymes needed for carbohydrate fermentation, this can be compensated for by the gut microbiota [77,78]. Bacteroidetes mainly produce acetate and propionate, and Firmicutes synthesize butyrate [79]. Abnormalities in cell morphology are observed in the absence of microorganisms, which can be partially recovered by SCFA supplementation, and SCFAs can be translocated from the gut mucosa to the circulation [80]. In addition, SCFAs suppress *Salmonella* growth [81], cancer [82], obesity [83], histone deacetylases [84,85], and autoimmunity [86].

We hypothesized that MDD is linked to human gut dysbiosis via LPS in the serum and brain. Altered intestinal epithelial levels of FABP2 and zonulin, regulators of endothelial and epithelial tight junctions, are linked to gut dysbiosis [87], and the serum FABP2 and zonulin levels were significantly restored after the flavonoid-rich orange intervention compared to the flavonoid-low orange intervention. Although the serum LPS level decreased after the flavonoid-rich treatment more than after the flavonoid-low treatment, the difference was not significant. However, *B. pullicaecorum* was significantly negatively correlated with FABP2 (r = −0.578, *p* < 0.05) and LPS (r = −0.730, *p* < 0.01) levels, which were elevated in patients with MDD and were decreased by the intervention-induced increases in *B. pullicaecorum*. In addition, SCFA levels were also correlated with changes in *B. pullicaecorum* abundance after the flavonoid-rich intervention. The propionic acid level (r = 0.655, *p* < 0.05) exhibited significant positive results and was significantly negatively correlated with the valeric acid level (r = −0.648, *p* < 0.05). We suppose that the increase in *B. pullicaecorum* abundance triggered by the flavonoid-rich orange intervention further increased the propionic acid level.

*B. pullicaecorum* is most closely related to *Clostridium* cluster IV, a butyrate-producing bacterium, and exposure to air for more than 3.5 h prevents its anaerobic growth [88]. In a randomized, double-blind, placebo-controlled crossover trial involving 30 healthy volunteers, *B. pullicaecorum* did not disrupt metabolic activity [89]. Suggesting its potential as a next-generation probiotic for the treatment of depression. There are some drawbacks to this study as well. A single 24-h recall does not represent habitual diet at an individual level. This survey is adequate for surveying estimated group mean intakes of diet [90]. Due to the bias caused by the uneven distribution of bacteria in feces after homogenization, microbiota analysis using fecal sampling cannot reliably show changes in gut microbiota [91]. However, this approach is inexpensive and provides a large amount of biomass for analysis. Another drawback is the unavoidable possibility of a change in the composition or metabolism of the microbiome following the consumption of a particular food during the study period.

## 5. Conclusions

In conclusion, orange flavonoid interventions significantly improved the HAMD-17, BDI, and CES-D scores in patients with MDD. Both interventions increased the abundance of *B. pullicaecorum*, which was correlated with serum LPS, FABP2, and valeric acid levels in fecal samples, which are negatively and positively correlated with propionic acid, one of the major SCFAs. This study was limited to being single-blind and lacking a placebo group. However, our findings suggest that in addition to increasing the abundance of *B. pullicaecorum* in patients with MDD, flavonoid-rich orange interventions have potential as next-generation probiotics for the treatment of depression.

## Figures and Tables

**Table 1:** Baseline characteristics of the participants.

Variables	FR Group (*n* = 20)	FL Group (*n* = 20)	*p*-Value
Sex, N (%)			
Male	6 (30)	6 (30)	1.000 ^‡^
Female	14 (70)	14 (70)	
Age, mean ± SD, year	24.70 ± 2.830	23.10 ± 2.594	0.070 ^†^
Residence type, N (%)			
Living alone	7 (35)	6 (30)	0.427 ^$^
With roommate	1 (5)	5 (25)	
With parents	9 (45)	6 (30)	
With parents and grandparents	2 (10)	1 (5)	
Others	1 (5)	2 (10)	
Household income, N (%)			
<1,000,000 won per month	1 (5)	4 (20)	0.227 ^$^
1,000,000–2,999,999 won per month	6 (30)	7 (35)	
3,000,000–4,999,999 won per month	5 (25)	1 (5)	
>5,000,000 won per month	8 (40)	8 (40)	
Regular physical activity, N (%)			
Yes	5 (25)	5 (25)	1.000 ^‡^
No	15 (75)	15 (75)	
Alcohol drinking, N (%)			
None	4 (20)	1 (5)	0.511 ^$^
Less than once a month	4 (20)	4 (20)	
Once a month	3 (15)	6 (30)	
2–4 times a month	3 (15)	5 (25)	
2–3 times a week	6 (30)	4 (20)	
Heavy drinking frequency, N (%)			
None	11 (55)	5 (25)	0.030 ^$^
Less than once a month	4 (20)	6 (30)	
Once a month	1 (5)	7 (35)	
Once a week	4 (20)	1 (5)	
Almost everyday	0	1 (5)	
Smoking, N (%)			
None	15 (75)	11 (55)	0.587 ^$^
Past smoking (no current smoking)	1 (5)	3 (15)	
Occasionally	1 (5)	1 (5)	
Always	3 (15)	5 (25)	
Stress, N (%)			
Too much	4 (20)	4 (20)	1.000 ^$^
Pretty much	12 (60)	11 (55)	
Little	4 (20)	5 (25)	

^†^ Student’s *t*-test, ^‡^ Chi-square test, ^$^ Fisher’s exact test, FR group: flavonoid-rich orange juice group; FL group: flavonoid-low orange cordial group.

**Table 2:** Anthropometric data and blood test and MDD screening results at baseline and 8 weeks after intervention.

Variables	FR Group (*n* = 20)		*p*-Value	FL Group (*n* = 20)		*p*-Value	Δ Group Comparison ^$^
	Baseline	After Intervention		Baseline	After Intervention		
	Mean ± SE			Mean ± SE			
Weight, kg	66.33 ± 3.09	67.53 ± 3.13	0.001 ^†^	59.70 ± 2.91	60.62 ± 2.95	0.007 ^†^	0.512
BMI, kg/m^2^	23.85 ± 0.80	24.29 ± 0.80	0.002 ^†^	22.00 ± 0.86	22.32 ± 0.87	0.016 ^†^	0.449
Percent body fat, %	31.42 ± 1.41	31.07 ± 1.40	0.737 ^‡^	28.48 ± 1.97	27.93 ± 1.81	0.338 ^‡^	0.754
Skeletal muscle mass, kg	24.87 ± 1.33	24.63 ± 1.43	0.376 ^‡^	23.16 ± 1.08	23.73 ± 1.09	0.285 ^‡^	0.346
SBP, mmHg	119.95 ± 3.39	120.00 ± 2.19	0.977 ^†^	113.21 ± 2.26	114.95 ± 2.14	0.524 ^†^	0.605
DBP, mmHg	76.47 ± 2.41	81.68 ± 1.83	0.064 ^†^	75.53 ± 2.75	77.21 ± 2.29	0.475 ^†^	0.321
BDNF	2033.61 ± 9.16	2157.80 ± 19.67	0.000 ^†^	2123.93 ± 16.13	2133.75 ± 9.87	0.670 ^†^	0.001
Serotonin, ng/mL	90.65 ± 10.98	111.24 ± 14.06	0.268 ^†^	94.64 ± 10.69	98.43 ± 14.78	0.846 ^†^	0.528
Folate, ng/mL	8.65 ± 1.24	9.03 ± 1.06	0.630 ^†^	7.50 ± 1.28	7.24 ± 0.84	0.970 ^‡^	0.646
Homocysteine, μmol/L	9.01 ± 0.92	8.31 ± 0.78	0.107 ^†^	8.73 ± 0.62	7.70 ± 0.51	0.090 ^†^	0.638
hs-CRP, mg/L	1.98 ± 0.69	1.03 ± 0.22	0.842 ^‡^	0.65 ± 1.67	2.21 ± 1.47	0.395 ^‡^	0.122
Vitamin B_12_, pg/Ml	624.70 ± 45.41	607.40 ± 54.55	0.639 ^†^	617.45 ± 87.21	609.15 ± 73.82	0.365 ^‡^	0.840
BDI-II	26.05 ± 1.38	14.10 ± 1.54	<0.001 ^†^	26.55 ± 1.18	17.05 ± 1.33	<0.001 ^†^	0.360
CES-D score	34.25 ± 2.06	15.90 ± 2.00	<0.001 ^†^	30.30 ± 1.82	18.15 ± 2.24	0.000 ^†^	0.110
HAMD-17	21.30 ± 1.11	8.30 ±1.29	<0.001 ^†^	21.80 ± 1.05	12.40 ± 1.32	<0.001 ^†^	0.114

BMI, body mass index; SBP, systolic blood pressure; DBP, diastolic blood pressure; BDNF, brain-derived neurotrophic factor; hs-CRP, high-sensitivity C-reactive protein; BDI-II, Beck Depression Inventory-II; CES-D, Center for Epidemiological Studies Depression Scale; HAMD, Hamilton Depression Rating Scale; ^†^ paired *t*-test, ^‡^ Wilcoxon signed-rank test, ^$^ Student’s *t*-test. FR group: flavonoid-rich orange juice group; FL group: flavonoid-low orange cordial group.

## Data Availability

The data that support the findings of this study are registered with the Clinical Research Information Service at https://cris.nih.go.kr/cris/index/index.do (accessed on 13 May 2021), reference number [KCT0006137], and protocol modifications were publicly reflected through CRIS.

## Supplementary Materials

The following supporting information can be downloaded at: https://www.mdpi.com/article/10.3390/nu15010145/s1, Figure S1: Comparison of phylotype coverage and phylogenetic diversity of the gut microbiota in before and after flavonoid-rich orange juice group and flavonoid-low orange cordial group intervention in depression. BFR; Before flavonoid-rich orange juice group, AFR; after flavonoid-rich orange juice group, BFL: before flavonoid-low orange cordial group, AFL: after flavonoid-low orange cordial group; Figure S2: Alpha diversity in terms of Faith’s phylogenetic diversity presented in different group comparisons; Table S1: Flavonoid content of FR-and FL group treatment drinks analyzed by LC-MS; Table S2: Energy and nutrition values of the FR and FL groups. Table S3: Results of the 24-h recall questionnaire regarding nutrient intake at baseline and 8 weeks after the intervention.

## References

[1] Kennis M., Gerritsen L., van Dalen M., Williams A., Cuijpers P., Bockting C. (2020). Prospective biomarkers of major depressive disorder: A systematic review and meta-analysis. Mol. Psychiatry.

[2] Malhi G.S., Mann J.J. (2018). Depression. Lancet.

[3] World Health Organization (2017). Depression and Other Common Mental Disorders: Global Health Estimates.

[4] Vlainić J.V., Šuran J., Vlainić T., Vukorep A.L. (2016). Probiotics as an Adjuvant Therapy in Major Depressive Disorder. Curr. Neuropharmacol..

[5] Cuijpers P., Sijbrandij M., Koole S.L., Andersson G., Beekman A.T., Reynolds C.F. (2014). Adding psychotherapy to antidepressant medication in depression and anxiety disorders: A meta-analysis. World Psychiatry.

[6] Lopez J.P., Kos A., Turecki G. (2018). Major depression and its treatment: MicroRNAs as peripheral biomarkers of diagnosis and treatment response. Curr. Opin. Psychiatry.

[7] Hritcu L., Ionita R., Postu P.A., Gupta G.K., Turkez H., Lima T.C., Carvalho C.U.S., de Sousa D.P. (2017). Antidepressant flavonoids and their relationship with oxidative stress. Oxidative Med. Cell. Longev..

[8] Jia S., Hou Y., Wang D., Zhao X. (2022). Flavonoids for depression and anxiety: A systematic review and meta-analysis. Crit. Rev. Food Sci. Nutr..

[9] Ali S., Corbi G., Maes M., Scapagnini G., Davinelli S. (2021). Exploring the impact of flavonoids on symptoms of depression: A systematic review and meta-analysis. Antioxidants.

[10] Ko Y.-H., Kim S.-K., Lee S.-Y., Jang C.-G. (2020). Flavonoids as therapeutic candidates for emotional disorders such as anxiety and depression. Arch. Pharmacal Res..

[11] Dayan E., Cohen L.G. (2011). Neuroplasticity subserving motor skill learning. Neuron.

[12] Duman R.S., Aghajanian G.K. (2012). Synaptic dysfunction in depression: Potential therapeutic targets. Science.

[13] Krystal J.H., Sanacora G., Duman R.S. (2013). Rapid-acting glutamatergic antidepressants: The path to ketamine and beyond. Biol. Psychiatry.

[14] Kraus C., Castrén E., Kasper S., Lanzenberger R. (2017). Serotonin and neuroplasticity—Links between molecular, functional and structural pathophysiology in depression. Neurosci. Biobehav. Rev..

[15] Castricum J., Birkenhager T.K., Kushner S.A., Elgersma Y., Tulen J.H.M. (2022). Cortical Inhibition and Plasticity in Major Depressive Disorder. Front. Psychiatry.

[16] Glinert A., Turjeman S., Elliott E., Koren O. (2022). Microbes, metabolites and (synaptic) malleability, oh my! The effect of the microbiome on synaptic plasticity. Biol. Rev. Camb. Philos. Soc..

[17] Martinowich K., Lu B. (2008). Interaction between BDNF and serotonin: Role in mood disorders. Neuropsychopharmacology.

[18] Khan A., Brown W.A. (2015). Antidepressants versus placebo in major depression: An overview. World Psychiatry.

[19] Jaffe D.H., Rive B., Denee T.R. (2019). The humanistic and economic burden of treatment-resistant depression in Europe: A cross-sectional study. BMC Psychiatry.

[20] Paris J. (2014). The mistreatment of major depressive disorder. Can. J. Psychiatry. Rev. Can. Psychiatr..

[21] Pennisi E. (2020). Meet the psychobiome. Science.

[22] Matarazzo I., Toniato E., Robuffo I. (2018). Psychobiome Feeding Mind: Polyphenolics in Depression and Anxiety. Curr. Top. Med. Chem..

[23] Tian P., Zou R., Song L., Zhang X., Jiang B., Wang G., Lee Y.K., Zhao J., Zhang H., Chen W. (2019). Ingestion of Bifidobacterium longum subspecies infantis strain CCFM687 regulated emotional behavior and the central BDNF pathway in chronic stress-induced depressive mice through reshaping the gut microbiota. Food Funct..

[24] Ait-Belgnaoui A., Durand H., Cartier C., Chaumaz G., Eutamene H., Ferrier L., Houdeau E., Fioramonti J., Bueno L., Theodorou V. (2012). Prevention of gut leakiness by a probiotic treatment leads to attenuated HPA response to an acute psychological stress in rats. Psychoneuroendocrinology.

[25] Bercik P., Verdu E.F., Foster J.A., Macri J., Potter M., Huang X., Malinowski P., Jackson W., Blennerhassett P., Neufeld K.A. (2010). Chronic gastrointestinal inflammation induces anxiety-like behavior and alters central nervous system biochemistry in mice. Gastroenterology.

[26] Strandwitz P. (2018). Neurotransmitter modulation by the gut microbiota. Brain Res..

[27] Ozogul F., Kuley E., Ozogul Y., Ozogul I. (2012). The Function of Lactic Acid Bacteria on Biogenic Amines Production by Food-Borne Pathogens in Arginine Decarboxylase Broth. Food Sci. Technol. Res..

[28] Stanaszek P.M., Snell J.F., Neill J.J.O. (1977). Isolation, extraction, and measurement of acetylcholine from *Lactobacillus plantarum*. Appl. Environ. Microbiol..

[29] Barrett E., Ross R.P., O’Toole P.W., Fitzgerald G.F., Stanton C. (2012). γ-Aminobutyric acid production by culturable bacteria from the human intestine. J. Appl. Microbiol..

[30] Tsavkelova E.A., Botvinko I.V., Kudrin V.S., Oleskin A.V. (2000). Detection of neurotransmitter amines in microorganisms with the use of high-performance liquid chromatography. Dokl. Biochem. Proc. Acad. Sci. USSR Biochem. Sect..

[31] Swann J.R., Want E.J., Geier F.M., Spagou K., Wilson I.D., Sidaway J.E., Nicholson J.K., Holmes E. (2011). Systemic gut microbial modulation of bile acid metabolism in host tissue compartments. Proc. Natl. Acad. Sci. USA.

[32] Quinn M., McMillin M., Galindo C., Frampton G., Pae H.Y., DeMorrow S. (2014). Bile acids permeabilize the blood brain barrier after bile duct ligation in rats via Rac1-dependent mechanisms. Dig. Liver Dis. Off. J. Ital. Soc. Gastroenterol. Ital. Assoc. Study Liver.

[33] Goyal D., Ali S.A., Singh R.K. (2021). Emerging role of gut microbiota in modulation of neuroinflammation and neurodegeneration with emphasis on Alzheimer’s disease. Prog. Neuro-Psychopharmacol. Biol. Psychiatry.

[34] Karakula-Juchnowicz H., Rog J., Juchnowicz D., Łoniewski I., Skonieczna-Żydecka K., Krukow P., Futyma-Jedrzejewska M., Kaczmarczyk M. (2019). The study evaluating the effect of probiotic supplementation on the mental status, inflammation, and intestinal barrier in major depressive disorder patients using gluten-free or gluten-containing diet (SANGUT study): A 12-week, randomized, double-blind, and placebo-controlled clinical study protocol. Nutr. J..

[35] Chang S.C., Cassidy A., Willett W.C., Rimm E.B., O’Reilly E.J., Okereke O.I. (2016). Dietary flavonoid intake and risk of incident depression in midlife and older women. Am. J. Clin. Nutr..

[36] Mihrshahi S., Dobson A.J., Mishra G.D. (2015). Fruit and vegetable consumption and prevalence and incidence of depressive symptoms in mid-age women: Results from the Australian longitudinal study on women’s health. Eur. J. Clin. Nutr..

[37] Pase M.P., Scholey A.B., Pipingas A., Kras M., Nolidin K., Gibbs A., Wesnes K., Stough C. (2013). Cocoa polyphenols enhance positive mood states but not cognitive performance: A randomized, placebo-controlled trial. J. Psychopharmacol..

[38] Caracci F., Harary J., Simkovic S., Pasinetti G.M. (2020). Grape-Derived Polyphenols Ameliorate Stress-Induced Depression by Regulating Synaptic Plasticity. J. Agric. Food Chem..

[39] Lamport D.J., Pal D., Macready A.L., Barbosa-Boucas S., Fletcher J.M., Williams C.M., Spencer J.P., Butler L.T. (2016). The effects of flavanone-rich citrus juice on cognitive function and cerebral blood flow: An acute, randomised, placebo-controlled cross-over trial in healthy, young adults. Br. J. Nutr..

[40] Kean R.J., Lamport D.J., Dodd G.F., Freeman J.E., Williams C.M., Ellis J.A., Butler L.T., Spencer J.P. (2015). Chronic consumption of flavanone-rich orange juice is associated with cognitive benefits: An 8-wk, randomized, double-blind, placebo-controlled trial in healthy older adults. Am. J. Clin. Nutr..

[41] Alharbi M.H., Lamport D.J., Dodd G.F., Saunders C., Harkness L., Butler L.T., Spencer J.P. (2016). Flavonoid-rich orange juice is associated with acute improvements in cognitive function in healthy middle-aged males. Eur. J. Nutr..

[42] Park K., Jaekal E., Yoon S., Lee S.-H., Choi K.-H. (2020). Diagnostic Utility and Psychometric Properties of the Beck Depression Inventory-II among Korean Adults. Front. Psychol..

[43] Helmreich I., Wagner S., König J., Kohnen R., Szegedi A., Hiemke C., Tadić A. (2015). Hamilton depression rating subscales to predict antidepressant treatment outcome in the early course of treatment. J. Affect. Disord..

[44] Radloff L.S. (1977). The CES-D Scale: A Self-Report Depression Scale for Research in the General Population. Appl. Psychol. Meas..

[45] Beck A.T., Steer R.A., Ball R., Ranieri W. (1996). Comparison of Beck Depression Inventories-IA and -II in psychiatric outpatients. J. Personal. Assess..

[46] Cho M.J., Kim K.H. (1998). Use of the Center for Epidemiologic Studies Depression (CES-D) Scale in Korea. J. Nerv. Ment. Dis..

[47] Lee K.H., Jae Y.M., Choi J.H., Jang S.H. (2014). Initial Response to Medication Predicts Early Improvement after 2 Weeks in Patients with Major Depressive Disorder. J. Korean Soc. Dep. Bip. Disord..

[48] Park M., Choi J., Lee H.J. (2020). Flavonoid-Rich Orange Juice Intake and Altered Gut Microbiome in Young Adults with Depressive Symptom: A Randomized Controlled Study. Nutrients.

[49] Estaki M., Jiang L., Bokulich N.A., McDonald D., González A., Kosciolek T., Martino C., Zhu Q., Birmingham A., Vázquez-Baeza Y. (2020). QIIME 2 Enables Comprehensive End-to-End Analysis of Diverse Microbiome Data and Comparative Studies with Publicly Available Data. Curr. Protoc. Bioinform..

[50] Callahan B.J., McMurdie P.J., Rosen M.J., Han A.W., Johnson A.J., Holmes S.P. (2016). DADA2: High-resolution sample inference from Illumina amplicon data. Nat. Methods.

[51] Katoh K., Kuma K.-I., Toh H., Miyata T. (2005). MAFFT version 5: Improvement in accuracy of multiple sequence alignment. Nucleic Acids Res.

[52] Price M.N., Dehal P.S., Arkin A.P. (2009). FastTree: Computing large minimum evolution trees with profiles instead of a distance matrix. Mol. Biol. Evol..

[53] McDonald D., Price M.N., Goodrich J., Nawrocki E.P., DeSantis T.Z., Probst A., Andersen G.L., Knight R., Hugenholtz P. (2012). An improved Greengenes taxonomy with explicit ranks for ecological and evolutionary analyses of bacteria and archaea. ISME J..

[54] Segata N., Izard J., Waldron L., Gevers D., Miropolsky L., Garrett W.S., Huttenhower C. (2011). Metagenomic biomarker discovery and explanation. Genome Biol..

[55] Grases G., Colom M.A., Sanchis P., Grases F. (2019). Possible relation between consumption of different food groups and depression. BMC Psychol..

[56] Ljungberg T., Bondza E., Lethin C. (2020). Evidence of the Importance of Dietary Habits Regarding Depressive Symptoms and Depression. Int. J. Environ. Res. Public Health.

[57] Li Y., Lv M.R., Wei Y.J., Sun L., Zhang J.X., Zhang H.G., Li B. (2017). Dietary patterns and depression risk: A meta-analysis. Psychiatry Res..

[58] Hintikka J., Tolmunen T., Honkalampi K., Haatainen K., Koivumaa-Honkanen H., Tanskanen A., Viinamäki H. (2005). Daily Tea Drinking Is Associated with a Low Level of Depressive Symptoms in the Finnish General Population. Eur. J. Epidemiol..

[59] Logan A.C. (2004). Omega-3 fatty acids and major depression: A primer for the mental health professional. Lipids Health Dis..

[60] Khalid S., Barfoot K.L., May G., Lamport D.J., Reynolds S.A., Williams C.M. (2017). Effects of Acute Blueberry Flavonoids on Mood in Children and Young Adults. Nutrients.

[61] Firoozabadi A., Kolouri S., Zarshenas M.M., Salehi A., Mosavat S.H., Dastgheib S.A. (2016). Efficacy of Nepeta Menthoides Boiss and Buhse Freeze-Dried Aqueous Extract on Anxiety of Patients with Depression: A Double-Blind Randomized Controlled Clinical Trial. Iran. J. Med. Sci..

[62] Macready A.L., Kennedy O.B., Ellis J.A., Williams C.M., Spencer J.P., Butler L.T. (2009). Flavonoids and cognitive function: A review of human randomized controlled trial studies and recommendations for future studies. Genes Nutr..

[63] Miller M.G., Shukitt-Hale B. (2012). Berry fruit enhances beneficial signaling in the brain. J. Agric. Food Chem..

[64] Pipingas A., Silberstein R.B., Vitetta L., Rooy C.V., Harris E.V., Young J.M., Frampton C.M., Sali A., Nastasi J. (2008). Improved cognitive performance after dietary supplementation with a Pinus radiata bark extract formulation. Phytother. Res. PTR.

[65] van Praag H., Lucero M.J., Yeo G.W., Stecker K., Heivand N., Zhao C., Yip E., Afanador M., Schroeter H., Hammerstone J. (2007). Plant-derived flavanol (-)epicatechin enhances angiogenesis and retention of spatial memory in mice. J. Neurosci. Off. J. Soc. Neurosci..

[66] Field D.T., Williams C.M., Butler L.T. (2011). Consumption of cocoa flavanols results in an acute improvement in visual and cognitive functions. Physiol. Behav..

[67] Scholey A.B., French S.J., Morris P.J., Kennedy D.O., Milne A.L., Haskell C.F. (2010). Consumption of cocoa flavanols results in acute improvements in mood and cognitive performance during sustained mental effort. J. Psychopharmacol..

[68] Cassidy A., Minihane A.M. (2017). The role of metabolism (and the microbiome) in defining the clinical efficacy of dietary flavonoids. Am. J. Clin. Nutr..

[69] Tomás-Barberán F.A., Selma M.V., Espín J.C. (2016). Interactions of gut microbiota with dietary polyphenols and consequences to human health. Curr. Opin. Clin. Nutr. Metab. Care.

[70] Cueva C., Gil-Sánchez I., Ayuda-Durán B., González-Manzano S., González-Paramás A.M., Santos-Buelga C., Bartolomé B., Moreno-Arribas M.V. (2017). An Integrated View of the Effects of Wine Polyphenols and Their Relevant Metabolites on Gut and Host Health. Molecules.

[71] O’Mahony S.M., Clarke G., Borre Y.E., Dinan T.G., Cryan J.F. (2015). Serotonin, tryptophan metabolism and the brain-gut-microbiome axis. Behav. Brain Res..

[72] Jenkins T.A., Nguyen J.C., Polglaze K.E., Bertrand P.P. (2016). Influence of Tryptophan and Serotonin on Mood and Cognition with a Possible Role of the Gut-Brain Axis. Nutrients.

[73] Dash S., Clarke G., Berk M., Jacka F.N. (2015). The gut microbiome and diet in psychiatry: Focus on depression. Curr. Opin. Psychiatry.

[74] Kowiański P., Lietzau G., Czuba E., Waśkow M., Steliga A., Moryś J. (2018). BDNF: A Key Factor with Multipotent Impact on Brain Signaling and Synaptic Plasticity. Cell. Mol. Neurobiol..

[75] Silva Y.P., Bernardi A., Frozza R.L. (2020). The Role of Short-Chain Fatty Acids from Gut Microbiota in Gut-Brain Communication. Front. Endocrinol..

[76] den Besten G., van Eunen K., Groen A.K., Venema K., Reijngoud D.J., Bakker B.M. (2013). The role of short-chain fatty acids in the interplay between diet, gut microbiota, and host energy metabolism. J. Lipid Res..

[77] Flint H.J., Duncan S.H., Scott K.P., Louis P. (2014). Links between diet, gut microbiota composition and gut metabolism. Proc. Nutr. Soc..

[78] Fernández J., Redondo-Blanco S., Gutiérrez-del-Río I., Miguélez E.M., Villar C.J., Lombó F. (2016). Colon microbiota fermentation of dietary prebiotics towards short-chain fatty acids and their roles as anti-inflammatory and antitumour agents: A review. J. Funct. Foods.

[79] Levy M., Thaiss C.A., Elinav E. (2016). Metabolites: Messengers between the microbiota and the immune system. Genes Dev..

[80] Erny D., Hrabě de Angelis A.L., Jaitin D., Wieghofer P., Staszewski O., David E., Keren-Shaul H., Mahlakoiv T., Jakobshagen K., Buch T. (2015). Host microbiota constantly control maturation and function of microglia in the CNS. Nat. Neurosci..

[81] El-Gedaily A., Paesold G., Chen C.Y., Guiney D.G., Krause M. (1997). Plasmid virulence gene expression induced by short-chain fatty acids in Salmonella dublin: Identification of rpoS-dependent and rpo-S-independent mechanisms. J. Bacteriol..

[82] Nakkarach A., Foo H.L., Song A.A.-L., Mutalib N.E.A., Nitisinprasert S., Withayagiat U. (2021). Anti-cancer and anti-inflammatory effects elicited by short chain fatty acids produced by Escherichia coli isolated from healthy human gut microbiota. Microb. Cell Factories.

[83] Lu Y., Fan C., Li P., Lu Y., Chang X., Qi K. (2016). Short Chain Fatty Acids Prevent High-fat-diet-induced Obesity in Mice by Regulating G Protein-coupled Receptors and Gut Microbiota. Sci. Rep..

[84] Li M., van Esch B.C.A.M., Henricks P.A.J., Folkerts G., Garssen J. (2018). The Anti-inflammatory Effects of Short Chain Fatty Acids on Lipopolysaccharide- or Tumor Necrosis Factor α-Stimulated Endothelial Cells via Activation of GPR41/43 and Inhibition of HDACs. Front. Pharmacol..

[85] Park J., Kim M., Kang S.G., Jannasch A.H., Cooper B., Patterson J., Kim C.H. (2015). Short-chain fatty acids induce both effector and regulatory T cells by suppression of histone deacetylases and regulation of the mTOR–S6K pathway. Mucosal Immunol..

[86] Luu M., Pautz S., Kohl V., Singh R., Romero R., Lucas S., Hofmann J., Raifer H., Vachharajani N., Carrascosa L.C. (2019). The short-chain fatty acid pentanoate suppresses autoimmunity by modulating the metabolic-epigenetic crosstalk in lymphocytes. Nat. Commun..

[87] Stevens B.R., Goel R., Seungbum K., Richards E.M., Holbert R.C., Pepine C.J., Raizada M.K. (2018). Increased human intestinal barrier permeability plasma biomarkers zonulin and FABP2 correlated with plasma LPS and altered gut microbiome in anxiety or depression. Gut.

[88] Eeckhaut V., Van Immerseel F., Teirlynck E., Pasmans F., Fievez V., Snauwaert C., Haesebrouck F., Ducatelle R., Louis P., Vandamme P. (2008). Butyricicoccus pullicaecorum gen. nov., sp. nov., an anaerobic, butyrate-producing bacterium isolated from the caecal content of a broiler chicken. Int. J. Syst. Evol. Microbiol..

[89] Boesmans L., Valles-Colomer M., Wang J., Eeckhaut V., Falony G., Ducatelle R., Immerseel F.V., Raes J., Verbeke K., Cotter P.D. (2018). Butyrate Producers as Potential Next-Generation Probiotics: Safety Assessment of the Administration of *Butyricicoccus pullicaecorum* to Healthy Volunteers. mSystems.

[90] Raina S.K. (2013). Limitations of 24-hour Recall Method: Micronutrient Intake and the Presence of the Metabolic Syndrome. N. Am. J. Med. Sci..

[91] Tang Q., Jin G., Wang G., Liu T., Liu X., Wang B., Cao H. (2020). Current sampling methods for gut microbiota: A call for more precise devices. Front. Cell. Infect. Microbiol..

