# Supplementary material for: Effects of Flavonoid-Rich Orange Juice Intervention on Major Depressive Disorder in Young Adults: A Randomized Controlled Trial
Source: Nutrients. 2022 Dec 28;15(1):145. doi: 10.3390/nu15010145 (PMC9823945; doi:10.3390/nu15010145)
Supplement: Supplementary file 1 [file nutrients-15-00145-s001.zip › nutrients-2088640-supplementary.pdf]

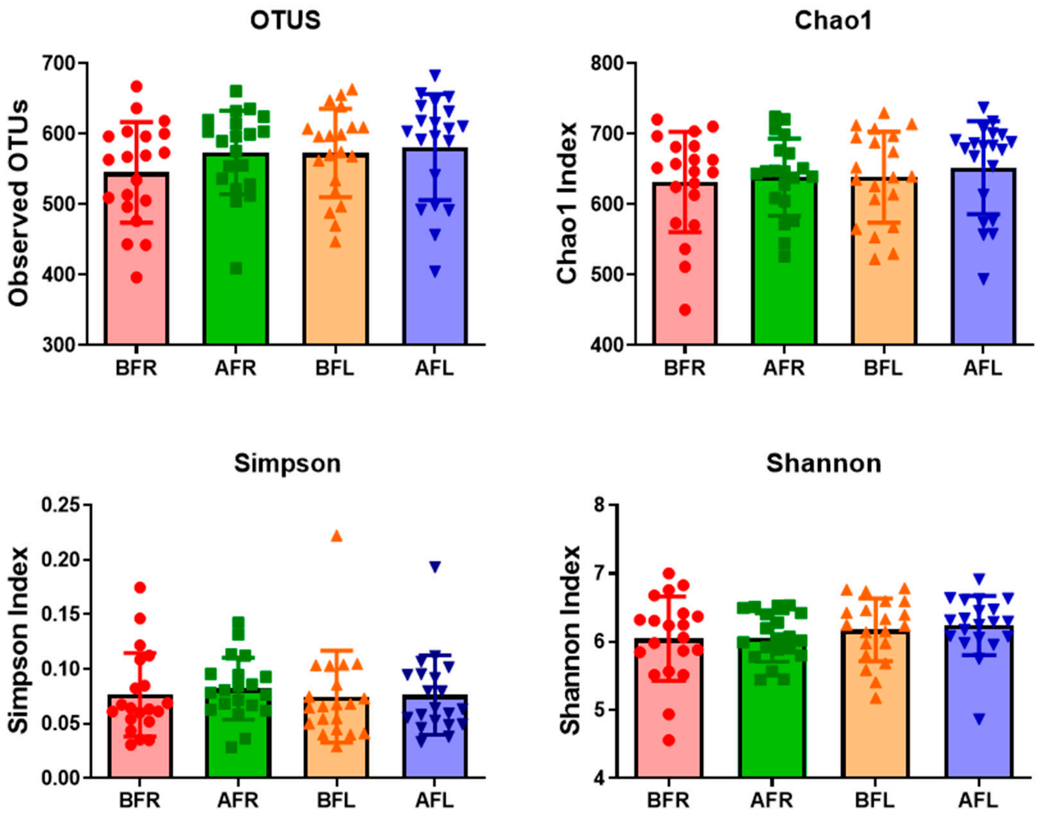

Supplementary Figure S1. Comparison of phylotype coverage and phylogenetic diversity of the gut microbiota in before and after flavonoid-rich orange juice group and flavonoid-low orange cordial group intervention in depression. BFR; Before flavonoid-rich orange juice group, AFR; after flavonoid-rich orange juice group, BFL: before flavonoid-low orange cordial group, AFL: after flavonoid-low orange cordial group.

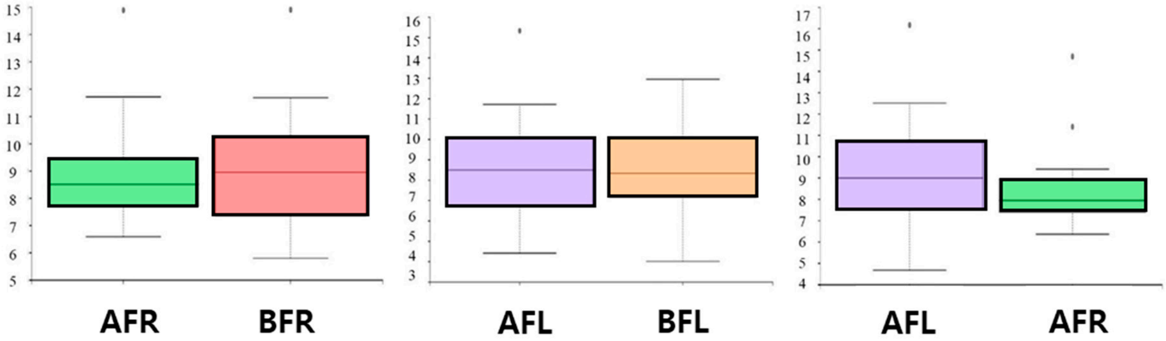

Supplementary Figure S2. Alpha diversity in terms of Faith's phylogenetic diversity presented in different group comparisons.

Supplementary Table S1. Flavonoid content of FR-and FL group treatment drinks analyzed by LC-MS

Aglycones	Peak No.	Individual flavonoids	MW	Fragment ions (m/z)	FR group treatment drinks	FL group treatment drinks
					(mg/100 g DW)	
Naringenin (m/z 273)	4	naringenin 7-O-rutinoside (narirutin)	580	603, 581, 435, 419, 273	28.3 ± 0.3	3.6 ± 0.1
	3	naringenin 7-O-rutinoside-4'-O-glucoside (narirutin 4'-O-glucoside)	742	765, 743, 581, 435, 273	10.4 ± 0.1	1.1 ± 0.0
Isosakuranetin (m/z 287)	7	isosakuranetin 7-O-rutinoside (didymin)	594	617, 595, 449, 433, 287	5.4 ± 0.3	1.0 ± 0.0
Hesperetin (m/z 303)	6	hesperetin 7-O-rutinoside (hesperidin)	610	633, 611, 465, 449, 303	74.2 ± 0.5	21.1 ± 0.6
Apigenin (m/z 271)	2	apigenin 6,8-di-C-glucoside(vicenin-2)	594	617, 595, 577, 559, 541, 475, 457, 439, 409	34.8 ± 0.2	1.4 ± 0.0
Luteolin (m/z 287)	1	luteolin 6,8-di-C-glucoside(lucenin-2)	610	633, 611, 593, 575, 557, 491, 473, 455, 425	2.3 ± 0.0	0.1 ± 0.0
Isorhamnetin (m/z 317)	5	isorhamnetin 3-O-rutinoside (narcissin)	624	647, 479, 317	2.5 ± 0.3	0.1 ± 0.0
		Total flavonoids			157.9 ± 1.4	28.4 ± 0.7

Supplementary Table S2. Energy and nutrition values of the FR and FL groups

Nutrients per 190 g (1 serve)	Treatment	
	FR group treatment drinks	FL group treatment drinks
	(% Daily Value)	
Energy, kcal	68	60
Total Carbohydrate, g	16 (6)	15 (15)
Sugar, g	14	15
Sodium, mg	0 (0)	25 (1)
Total fat, g	0 (0)	0 (0)
Saturated fat, g	0 (0)	0 (0)
Trans fat, g	0 (0)	0 (0)
Cholesterol, mg	0 (0)	0 (0)
Protein, g	0 (0)	0 (0)

Supplementary Table S3. Results of the 24-h recall questionnaire regarding nutrient intake at baseline and 8 weeks after the intervention

Variables	FR group (n=20)		p-value†	FL group (n=20)		p-value†	Δ group comparison ¥
	Baseline	After intervention		Baseline	After intervention		
	Mean ± SD			Mean ± SD			
Energy, kcal	1387.47 ± 639.23	2505.12 ± 664.98	<0.0001†	1597.74 ± 994.39	1943.60 ± 595.80	0.180†	0.013
Carbohydrate, g	187.65 ± 77.68	462.74 ± 104.60	<0.0001†	190.90 ± 116.71	286.05 ± 66.24	0.003†	<0.0001
Protein, g	58.96 ± 40.14	69.12 ± 22.35	0.044‡	56.73 ± 34.38	65.31 ± 25.25	0.386†	0.897
Fat, g	43.35 ± 29.56	53.07 ± 31.64	0.094†	59.97 ± 45.79	59.13 ± 35.54	0.951†	0.471
Calcium, mg	334.39 ± 227.00	1059.91 ± 261.48	<0.0001†	395.13 ± 246.44	483.02 ± 285.90	0.316†	<0.0001
Phosphate, mg	765.87 ± 391.88	1092.84 ± 291.01	0.004†	811.19 ± 429.41	982.73 ± 353.45	0.213†	0.355
Iron, mg	9.72 ± 5.61	17.81 ± 22.29	0.010‡	9.38 ± 5.78	11.79 ± 4.35	0.165†	0.237
Sodium, mg	2554.95 ± 1372.87	2618.62 ± 1297.67	0.873†	2569.02 ± 1589.40	3158.23 ± 1708.51	0.151†	0.351
Potassium, mg	1700.54 ± 891.81	4493.75 ± 1117.01	<0.0001†	1540.44 ± 897.99	2806.18 ± 696.42	<0.0001†	<0.0001
Vitamin A, μg RE	246.80 ± 165.95	404.39 ± 171.24	0.006†	252.47 ± 163.12	327.46 ± 262.37	0.285†	0.337
β-carotene, μg	1359.47 ± 852.39	3771.10 ± 1976.07	<0.0001†	1449.93 ± 1408.89	1928.38 ± 1378.03	0.248†	0.003
Thiamin, mg	1.04 ± 0.50	3.77 ± 1.05	<0.0001‡	1.39 ± 1.37±	1.82 ± 0.81	0.033‡	<0.0001
Riboflavin, mg	0.98 ± 0.61	1.42 ± 0.49	0.024†	1.17 ± 0.81	1.40 ± 0.79	0.402†	0.527
Niacin, mg	11.63 ± 7.64	17.54 ± 6.27	<0.0001†	9.49 ± 7.50	11.61 ± 4.79	0.289†	0.121
Pyridoxine, mg	1.08 ± 0.67	2.35 ± 0.57	<0.0001	1.02 ± 0.97	1.71 ± 0.50	0.004†	0.035
Folate, μg	256.56 ± 161.08	1410.71 ± 308.25	<0.0001‡	232.37 ± 133.74	657.03 ± 163.89	<0.0001†	<0.0001
Vitamin B_{12}, μg	6.10 ± 5.39	4.45 ± 4.05	0.299†	4.40 ± 4.50	6.43 ± 4.38	0.128†	0.074
Vitamin C, mg	37.47 ± 26.72	1060.24 ± 221.33	<0.0001‡	44.54 ± 76.52	233.87 ± 93.79	<0.0001‡	<0.0001

†Paired t-test, ‡Wilcoxon signed rank test, ¥ Student t-test.
